# Supplementary material for: Core collection construction and genetic diversity analysis of tea plant (Camellia sinensis [L:] O. Kuntze) accessions in Huangshan city using SSR markers
Source: PLoS One. 2025 Apr 24;20(4):e0322209. doi: 10.1371/journal.pone.0322209 (PMC12021250; doi:10.1371/journal.pone.0322209)
Supplement: S1 Table — (DOCX) [file pone.0322209.s001.docx]

**S1 Table.** **Geographic distribution of 292 collected tea accessions.**

| **Sample name** | **Sample size** | **Sample type** | **Geographical location** | **Regions** | **Longitude and latitude** |
| --- | --- | --- | --- | --- | --- |
| HS01~HS04 | 4 | landraces | Tangkou town | Huangshan district | E 118°10′57″,N 30°4′15″ |
| HS05~HS08 | 4 | landraces | Tanjiaqiao town | Huangshan district | E 118°16′16″,N 30°9′52″ |
| HS09~HS12 | 4 | landraces | Xianyuan town | Huangshan district | E 118°12′8″,N 30°17′43″ |
| HS13~HS16 | 4 | landraces | Gengcheng town | Huangshan district | E 118°8′53″,N 30°14′17″ |
| HS17~HS20 | 4 | landraces | Gantang town | Huangshan district | E 118°7′24″,N 30°17′40″ |
| HS21~HS36 | 16 | landraces | Wushi town | Huangshan district | E 117°59′47″,N 30°15′52″ |
| HS37~HS44 | 8 | landraces | Jiaocun town | Huangshan district | E 118°4′18″,N 30°11′16″ |
| HS45~HS50 | 6 | landraces | Taiping lake town | Huangshan district | E 118°5′15″,N 30°23′29″ |
| HZ01~HZ10 | 10 | landraces | Fuxi township | Huizhou district | E 118°13′25″,N 29°59′50″ |
| HZ11~HZ20 | 10 | landraces | Qiashe township | Huizhou district | E 118°12′18″,N 29°55′59″ |
| HZ21~HZ28 | 8 | landraces | Qiankou town | Huizhou district | E 118°17′44″,N 29°52′16″ |
| HZ29~HZ36 | 8 | landraces | Yangcun township | Huizhou district | E 118°10′38″,N 29°58′42″ |
| HZ37~HZ42 | 6 | landraces | Chenkan town | Huizhou district | E 118°16′45″,N 29°54′55″ |
| QM01~QM04 | 4 | landraces | Anling town | Qimen county | E 117°37′32″,N 30°2′31″ |
| QM05~QM08 | 4 | landraces | Qishan town | Qimen county | E 117°43′33″,N 29°50′54″ |
| QM09~QM11 | 3 | landraces | Fufeng town | Qimen county | E 117°50′16″,N 29°44′46″ |
| QM12~QM13 | 2 | landraces | Pingli town | Qimen county | E 117°36′49″,N 29°43′53″ |
| QM14~QM15 | 2 | landraces | Qihong township | Qimen county | E 117°40′34″,N 29°41′23″ |
| QM16~QM17 | 2 | landraces | Xiaolukou town | Qimen county | E 117°36′43″,N 29°51′29″ |
| QM18~QM20 | 3 | landraces | Zhukou township | Qimen county | E 117°29′19″,N 29°51′7″ |
| QM22~QM25 | 4 | landraces | Likou town | Qimen county | E 117°30′45″,N 29°53′41″ |
| QM26~QM28 | 3 | landraces | Guxi township | Qimen county | E 117°34′56″,N 29°56′41″ |
| QM29~QM30 | 2 | landraces | Ruokeng township | Qimen county | E 117°23′9″,N29°56′43″ |
| QM31~QM33 | 3 | landraces | Xinan town | Qimen county | E 117°17′2″,N 29°52′37″ |
| QM34~QM35 | 2 | landraces | Datan township | Qimen county | E 117°45′26″,N 29°56′35″ |
| QM36~QM37 | 2 | landraces | Jinzipai town | Qimen county | E 117°48′45″,N 29°50′40″ |
| QM38~QM40 | 3 | landraces | Luxi township | Qimen county | E 117°30′47″,N 29°42′17″ |
| QM41~QM43 | 3 | landraces | Tafang town | Qimen county | E 117°39′56″,N 29°46′56″ |
| QM44~QM45 | 2 | landraces | Rongkou township | Qimen county | E 117°33′5″,N 29°44′28″ |
| SX01~SX06 | 6 | landraces | Jiekou town | She county | E 118°43′38″,N 29°44′52″ |
| SX07~SX08 | 2 | landraces | Changxi township | She county | E 118°38′58″,N 29°54′33″ |
| SX09~SX16 | 8 | landraces | Wangcun town | She county | E 118°23′59″,N 29°45′27″ |
| SX17~SX25 | 9 | landraces | Qizili town | She county | E 118°44′30″,N 29°59′55″ |
| SX26~SX36 | 11 | landraces | Huangtian township | She county | E 118°38′57″,N 29°43′7″ |
| SX37~SX40 | 4 | landraces | Chakou town | She county | E 118°42′35″,N 29°53′7″ |
| SX41~SX47 | 8 | landraces | Shaolian township | She county | E 118°28′7″,N 29°42′11″ |
| SX48~SX49 | 2 | landraces | Xiaochuan township | She county | E 118°39′19″,N 29°48′4″ |
| SX50~SX54 | 5 | landraces | Changgai township | She county | E 118°31′47″,N 29°39′28″ |
| SX55~SX58 | 4 | landraces | Kengkou town | She county | E 118°31′55″,N 29°48′29″ |
| SX59~SX62 | 4 | landraces | Sencun township | She county | E 118°29′7″,N 29°45′17″ |
| SX63~SX66 | 4 | landraces | Xiongcun town | She county | E 118°26′28″,N 29°49′12″ |
| SX67~SX69 | 3 | landraces | Wuyang township | She county | E 118°39′28″,N 29°50′48″ |
| XN01~XN01 | 1 | landraces | Haiyang town | Xiuning county | E 118°10′28″,N 29°47′30″ |
| XN02~XN03 | 2 | landraces | Lingnan township | Xiuning county | E 118°8′48″,N 29°25′12″ |
| XN04~XN04 | 1 | landraces | Shandou township | Xiuning county | E 118°9′56″,N 29°33′30″ |
| XN05~XN07 | 3 | landraces | Shangshan town | Xiuning county | E 118°13′22″,N 29°39′37″ |
| XN08~XN08 | 1 | landraces | Wanan town | Xiuning county | E 118°12′42″,N 29°47′25″ |
| XN09~XN10 | 2 | landraces | Wucheng town | Xiuning county | E 118°11′9″,N 29°36′7″ |
| XN11~XN13 | 3 | landraces | Banqiao township | Xiuning county | E 117°57′10″,N 29°36′26″ |
| XN14~XN15 | 2 | landraces | Yuetanhu town | Xiuning county | E 118°4′35″,N 29°40′13″ |
| XN16~XN21 | 6 | landraces | Hecheng township | Xiuning county | E 117°43′50″,N 29°39′46″ |
| XN22~XN24 | 3 | landraces | Liukou town | Xiuning county | E 117°47′22″,N 29°42′12″ |
| XN25~XN28 | 4 | landraces | Wangcun town | Xiuning county | E 117°49′12″,N 29°38′16″ |
| XN29~XN32 | 4 | landraces | Xikou town | Xiuning county | E 118°0′44″,N 29°41′10″ |
| XN33~XN35 | 3 | landraces | Lantian town | Xiuning county | E 118°5′56″,N 29°54′21″ |
| XN36~XN37 | 2 | landraces | Longtian township | Xiuning county | E 118°18′33″,N 29°25′37″ |
| XN38~XN39 | 2 | landraces | Donglinxi town | Xiuning county | E 118°17′58″,N 29°40′23″ |
| XN40~XN41 | 2 | landraces | Yucun township | Xiuning county | E 118°21′53″,N 29°39′50″ |
| XN42~XN43 | 2 | landraces | Yuanfang township | Xiuning county | E 118°19′23″,N 29°36′15″ |
| XN44~XN45 | 2 | landraces | Qiyunshan town | Xiuning county | E 118°2′11″,N 29°48′33″ |
| XN46~XN47 | 2 | landraces | Baiji township | Xiuning county | E 118°23′55″,N 29°31′57″ |
| XN48~XN48 | 1 | landraces | Weiqiao township | Xiuning county | E 118°5′19″,N 29°46′13″ |
| YX01~YX04 | 4 | landraces | Yuting town | Yi county | E 117°58′50″,N 29°50′15″ |
| YX05~YX10 | 6 | landraces | Hongcun town | Yi county | E 117°59′24″,N 30°0′9″ |
| YX11~YX17 | 7 | landraces | Hongxing township | Yi county | E 117°49′19″,N 30°0′49″ |
| YX18~YX21 | 4 | landraces | Xidi town | Yi county | E 117°59′46″,N 29°54′12″ |
| YX22~YX25 | 4 | landraces | Kecun town | Yi county | E 117°45′0″,N 30°6′39″ |
| YX26~YX30 | 5 | landraces | Meixi township | Yi county | E 117°48′4″,N 30°4′37″ |
| YX31~YX34 | 4 | landraces | Biyang town | Yi county | E 117°55′17″,N 29°55′33″ |
| Wancha No.4 | 1 | cultivar | Ruokeng township | Qimen county | E 117°23′9″,N29°56′43″ |
| Wancha No.5 | 1 | cultivar | Ruokeng township | Qimen county | E 117°23′9″,N29°56′43″ |
| Anhui No.1 | 1 | cultivar | Qishan town | Qimen county | E 117°43′33″,N 29°50′54″ |
| Anhui No.3 | 1 | cultivar | Qishan town | Qimen county | E 117°43′33″,N 29°50′54″ |
| Caoxi No.1 | 1 | cultivar | Fuxi township | Huizhou district | E 118°13′25″,N 29°59′50″ |
