# Supplementary material for: Core collection construction and genetic diversity analysis of tea plant (Camellia sinensis [L:] O. Kuntze) accessions in Huangshan city using SSR markers
Source: PLoS One. 2025 Apr 24;20(4):e0322209. doi: 10.1371/journal.pone.0322209 (PMC12021250; doi:10.1371/journal.pone.0322209)
Supplement: S2 Table — (DOCX) [file pone.0322209.s002.docx]

**S2 Table Information of the reserved accessions.**

| **Location** | **Reserved accessions** | **Percentage (%)** |
| --- | --- | --- |
| QM | AH01/WC05/QM01/QM04/QM05/QM11/QM17/QM22/QM23/QM24/QM25/QM29/QM32/QM33/QM34/QM38/QM40/QM43/QM45 | 18.6 |
| SX | SX08/SX10/SX13/SX15/SX16/SX18/SX19/SX22/SX24/SX27/SX28/SX29/SX37/SX43/SX55/SX57/SX58/SX61/SX64/SX66/SX67/SX68 | 21.6 |
| XN | XN03/XN05/XN10/XN12/XN13/XN14/XN16/XN18/XN24/XN25/XN35/XN39/XN41/XN45 | 13.7 |
| YX | YX04/YX07/YX10/YX12/YX13/YX14/YX15/YX17/YX21/YX25/YX26/YX28/YX29/YX30 | 13.7 |
| HS | HS03/HS08/HS09/HS10/HS13/HS14/HS15/HS16/HS17/HS19/HS29/HS35/HS37/HS39/HS42/HS44/HS47/HS50 | 17.6 |
| HZ | HZ02/HZ03/HZ07/HZ08/HZ10/HZ16/HZ18/HZ21/HZ22/HZ23/HZ25/HZ27/HZ31/HZ37/HZ39 | 14.7 |
| HS: Huangshan district, HZ: Huizhou district, QM: Qimen county, SX: She county, XN: Xiuning county, YX: Yi county, Percentage (%), the percentage of the core collections quantity. | | |
