# Supplementary material for: Core collection construction and genetic diversity analysis of tea plant (Camellia sinensis [L:] O. Kuntze) accessions in Huangshan city using SSR markers
Source: PLoS One. 2025 Apr 24;20(4):e0322209. doi: 10.1371/journal.pone.0322209 (PMC12021250; doi:10.1371/journal.pone.0322209)
Supplement: Attachment — Submitted filename: Review.docx [file pone.0322209.s004.docx]

Dear Editor,

I have thoroughly read the article, and my detailed report is provided below.

This study presents a well-structured and comprehensive analysis of tea genetic diversity, particularly in the Anhui region. The introduction effectively highlights the challenges associated with the decline of local landraces and the importance of conservation efforts. The materials and methods section is detailed and thorough, providing enough information for replication, which enhances the study's reliability. The results offer valuable insights, particularly the high genetic diversity in She County and the division of population structure into two distinct groups, supported by multiple analytical approaches. The creation of a core collection retaining 82.62% of allele richness is commendable, though further explanation on core collection selection would strengthen the findings. Overall, this study makes a valuable contribution to tea genetic resource conservation.

**Introduction part:** This study's introduction provides a well-structured and comprehensive context on the origins, genetic diversity, and conservation of tea. The authors highlight key issues regarding tea production in the Anhui region, particularly the decline of local landraces and the widespread use of clonal varieties. They effectively address the challenges of preserving genetic resources and emphasize the importance of conservation efforts, such as the establishment of the Anhui Landraces Germplasm Tea Repository. Overall, this paper addresses a gap in the conservation of tea diversity, but making certain sections more accessible would enhance its impact.

**Materials and Methods section:** The methodology presented in the study is sufficiently detailed and comprehensive, covering all aspects necessary for replicating the experiments. The authors clearly describe the selection of SSR markers, DNA extraction, and PCR amplification processes, which enhances the reproducibility of the experiment. Overall, this section is comprehensive and well-suited to the study's goal of genetic analysis.

**Results and Discussion sections:** This study offers important insights into the genetic diversity of tea accessions, with SSR markers showing strong discriminatory capability and high levels of polymorphism. It effectively summarizes key findings and highlights the genetic diversity of tea landrace varieties. A significant observation is the high genetic diversity in She County, where the slower adoption of clonal tea varieties has helped preserve local landraces. The division of the population structure into two distinct groups, supported by Neighbor-Joining, PCoA, and Structure analyses, reflects a comprehensive assessment of the genetic data. The core collection, retaining 82.62% of allele richness, demonstrates that it was constructed efficiently, maintaining overall high genetic diversity. However, additional explanatory analysis and comparison regarding the selection of the core collection would further reinforce the conclusions. In summary, this research makes a valuable contribution to the conservation and understanding of tea genetic resources.
